# Associations between skeletal muscle energetics and accelerometry‐based performance fatigability: Study of Muscle, Mobility and Aging

**DOI:** 10.1111/acel.14015

**Published:** 2023-10-16

**Authors:** Yujia (Susanna) Qiao, Adam J. Santanasto, Paul M. Coen, Peggy M. Cawthon, Steven R. Cummings, Daniel E. Forman, Bret H. Goodpaster, Jaroslaw Harezlak, Marquis Hawkins, Stephen B. Kritchevsky, Barbara J. Nicklas, Frederico G. S. Toledo, Pamela E. Toto, Anne B. Newman, Nancy W. Glynn

**Affiliations:** ^1^ Department of Epidemiology, School of Public Health University of Pittsburgh Pittsburgh Pennsylvania USA; ^2^ AdventHealth, Translational Research Institute Orlando Florida USA; ^3^ San Francisco Coordinating Center California Pacific Medical Center Research Institute San Francisco California USA; ^4^ Department of Epidemiology and Biostatistics, School of Medicine University of California San Francisco San Francisco California USA; ^5^ Department of Medicine (Geriatrics and Cardiology) University of Pittsburgh, and Geriatrics, Research, Education, and Clinical Center (GRECC), VA Pittsburgh Healthcare System Pittsburgh Pennsylvania USA; ^6^ Department of Epidemiology and Biostatistics, School of Public Health‐Bloomington Indiana University Bloomington Indiana USA; ^7^ Gerontology and Geriatric Medicine Wake Forest School of Medicine Winston‐Salem North Carolina USA; ^8^ Division of Endocrinology and Metabolism, Department of Medicine University of Pittsburgh Pittsburgh Pennsylvania USA; ^9^ Department of Occupational Therapy University of Pittsburgh School of Health and Rehabilitation Sciences Pittsburgh Pennsylvania USA

**Keywords:** aging, fatigability, fatigue, gait, mitochondria

## Abstract

Performance fatigability is typically experienced as insufficient energy to complete daily physical tasks, particularly with advancing age, often progressing toward dependency. Thus, understanding the etiology of performance fatigability, especially cellular‐level biological mechanisms, may help to delay the onset of mobility disability. We hypothesized that skeletal muscle energetics may be important contributors to performance fatigability. Participants in the Study of Muscle, Mobility and Aging completed a usual‐paced 400‐m walk wearing a wrist‐worn ActiGraph GT9X to derive the Pittsburgh Performance Fatigability Index (PPFI, higher scores = more severe fatigability) that quantifies percent decline in individual cadence‐versus‐time trajectory from their maximal cadence. Complex I&II‐supported maximal oxidative phosphorylation (max OXPHOS) and complex I&II‐supported electron transfer system (max ETS) were quantified ex vivo using high‐resolution respirometry in permeabilized fiber bundles from vastus lateralis muscle biopsies. Maximal adenosine triphosphate production (ATP_max_) was assessed in vivo by ^31^P magnetic resonance spectroscopy. We conducted tobit regressions to examine associations of max OXPHOS, max ETS, and ATP_max_ with PPFI, adjusting for technician/site, demographic characteristics, and total activity count over 7‐day free‐living among older adults (*N* = 795, 70–94 years, 58% women) with complete PPFI scores and ≥1 energetics measure. Median PPFI score was 1.4% [25th–75th percentile: 0%–2.9%]. After full adjustment, each 1 standard deviation lower max OXPHOS, max ETS, and ATP_max_ were associated with 0.55 (95% CI: 0.26–0.84), 0.39 (95% CI: 0.09–0.70), and 0.54 (95% CI: 0.27–0.81) higher PPFI score, respectively. Our findings suggested that therapeutics targeting muscle energetics may potentially mitigate fatigability and lessen susceptibility to disability among older adults.

## Graphical abstract

Among 795 older adults from the Study of Muscle, Mobility and Aging, we found that each 1 standard deviation lower max OXPHOS, max ETS, and ATPmax were associated with 0.55 (95% CI: 0.26–0.84), 0.39 (95% CI: 0.09–0.70), and 0.54 (95% CI: 0.27–0.81) higher PPFI score, respectively. Our findings suggested that therapeutics targeting muscle energetics may potentially mitigate fatigability and lessen susceptibility to disability among older adults.

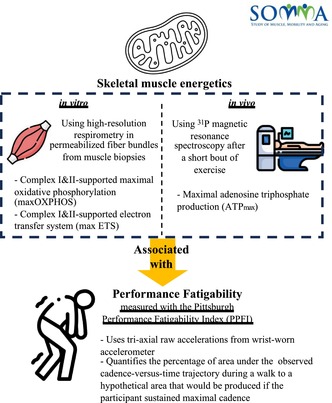

**Abbreviations**
ANOVA: analysis of variance
ATP: adenosine triphosphate
BMI: body mass index
ETS: electronic transfer system
OXPHOS: oxidative phosphorylation
PCr: phosphocreatine
PPFI: pittsburgh performance fatigability index
ROS: reactive oxygen species
SD: standard deviation
SOMMA: Study of Muscle, Mobility and Aging

## 1 INTRODUCTION

Skeletal muscle mitochondria produce the energy needed for muscle contraction and are the key energetic drivers of locomotion and mobility (Coen et al., 2013; Mau et al., 2022; Santanasto et al., 2016; Tian et al., 2023). With aging, skeletal muscle energetics decline, particularly for oxidative phosphorylation (OXPHOS) and adenosine triphosphate (ATP) generation capacity (Gonzalez‐Freire et al., 2015; Short et al., 2005). Meanwhile, performance fatigability, an energy disorder, worsens and manifests as insufficient energy to complete daily physical tasks (Alexander et al., 2010; Eldadah, 2010). In the aging literature, performance fatigability is defined as a decrement in performance during a standardized physical task or activity (Schrack et al., 2020; Van Geel et al., 2020) and has been associated with functional limitations/mobility decline (Murphy et al., 2017; Simonsick et al., 2014) and frailty (Schnelle et al., 2012) among older adults. Given the importance of skeletal muscle in physical performance and mobility (Gonzalez‐Freire et al., 2018; Lanza & Nair, 2010), better understanding of skeletal muscle energetics and performance fatigability may provide valuable insights to identify potential mitochondrial therapeutic targets that prevent fatigability and promote healthy aging.

To our knowledge, only two studies have directly examined the associations between muscle energetics and fatigability (Liu et al., 2021; Santanasto et al., 2015). However, they focused on perceived fatigability, a similar, but distinct, concept from performance fatigability (Loy et al., 2017; Simonsick et al., 2014). Studies by Santanasto et al. (2015) and Liu et al. (2021) both concluded that poorer skeletal muscle maximal ATP production (ATP_max_), as measured with ^31^P magnetic resonance spectroscopy (MRS), was associated with more severe perceived fatigability. Extending this work by measuring performance fatigability objectively using accelerometry plus measuring mitochondrial respiration in addition to ATP production would capture skeletal muscle energetics more comprehensively. Specifically, high‐resolution respirometry has been recognized as the gold standard to measure OXPHOS in permeabilized muscle fibers from biopsies (Gnaiger, 2001), which provides mechanistic component of mitochondrial function that complements and extends assessments of ATP synthesis (i.e., ATP_max_). Additionally, high‐resolution respirometry also assesses electron transfer system (ETS) capacity, which is quantified as the maximum oxygen flux stimulated by non‐coupled respiration with a short circuit of the proton cycle across the inner mitochondria membrane. By analyzing both respirometry and ATP production, we can further explore mechanical similarities and differences of maximal mitochondrial function capacity versus capacity under pathological conditions in relation to fatigability, which may further inform us on the optimal lifestyle intervention modality to mitigate fatigability severity.

Collectively, existing evidence provides a strong scientific premise for examining associations between skeletal muscle energetics and performance fatigability. The Study of Muscle, Mobility and Aging (SOMMA) included assessments of both skeletal muscle respirometry and ATP production in a large prospective cohort of older adults, as well as objectively measured performance fatigability called the Pittsburgh Performance Fatigability Index (PPFI). We hypothesized that lower Complex I&II‐supported maximal OXPHOS, Complex I&II‐supported ETS, and ATP_max_ would be significantly associated with higher PFFI score.

## 2 METHOD

### 2.1 Study sample

A total of 879 community‐dwelling women and men (age ≥70 years) with gait speed of ≥0.6 m/s during a 4‐m walk and willing/eligible to have MRS and a skeletal muscle biopsy were enrolled in SOMMA (http://sommaonline.ucsf.edu) at two academic clinical centers (University of Pittsburgh and Wake Forest University School of Medicine) from April 2019 to December 2021 (Cummings et al., 2023). Participants were excluded if they reported an inability to walk one‐quarter of a mile or climb a flight of stairs; had body mass index (BMI) ≥40 kg/m^2^; had an active malignancy or dementia; or had any medical contraindication to biopsy or MR. Briefly, as a prospective, longitudinal ongoing cohort study, SOMMA aims to clarify the constellation of muscle properties that most strongly predict mobility disability, decline in fitness, walking speed, and muscle mass as people age, which might be targets for new interventions. The baseline visit generally included 3 days of assessments: Day 1—usual‐paced 400‐m walk and general clinic measures, Day 2—magnetic resonance imaging/spectroscopy, and Day 3—skeletal muscle biopsy. Full details of the study design and timing of clinical measurements have been published (Cummings et al., 2023). All participants provided written informed consent, and SOMMA was approved by the WIRB‐Copernicus Group Institutional Review Board (WCG IRB) as the single IRB for all participating sites.

### 2.2 Skeletal muscle energetics

#### 2.2.1 Skeletal muscle biopsy collection and processing

The skeletal muscle biopsy was obtained from the right side medial vastus lateralis after an 12‐h fast and limited strenuous exercise for 48 h before the procedure. Participants also rested in a supine position for at least 15 min prior to the biopsy. Skeletal muscle biopsy specimens were obtained under local anesthesia (1% or 2% lidocaine), ~15 cm above the patella using a 5 or 6 mm Bergstrom‐style core biopsy needle with suction. Muscle tissue was immediately blotted dry and trimmed of visible adipose and connective tissue. A separate portion (~20 mg) of the biopsy specimen was placed in a relaxing and biopsy‐preserving solution for high‐resolution respirometry, as previously described (Coen et al., 2013). Myofiber bundles (~2–3 mg) were prepared by gently teasing apart the fibers using fine‐point tweezers. Then, myofiber bundles were chemically permeabilized for 30 min using saponin and were washed twice before determining their wet weight on an analytical balance (Mettler Toledo). Details on fiber bundle preparation can be found elsewhere (Coen et al., 2015).

#### 2.2.2 Mitochondrial respiration

After weighing, the permeabilized fiber bundles were placed into the respirometer chambers of an Oxygraph‐2K (O2K, Oroboros Instruments). Assays were run at 37°C in a specific O_2_ concentration range (400–200 μM) in respiratory chambers. In the assay protocol, the following substrates were added in sequential steps to assess Complex I‐ and II‐supported maximal OXPHOS at state 3 respiration (max OXPHOS): pyruvate (5 mM) and malate (2 mM), ADP (4.2 mM) and Cytochrome *c* (10 μM), glutamate (10 mM), and succinate (10 mM). Then, a titration of the uncoupler FCCP was further added to determine Complex I and II‐supported ETS (max ETS). Any sample with the integrity of the outer mitochondrial membrane, as tested with Cytochrome *c*, greater than 15% was omitted from the analysis. Steady‐state oxygen flux was normalized to wet weight of the fiber bundle using Datlab 7.4 software. In SOMMA, the mean coefficient of variation for duplicates of max OXPHOS measurement was 11.5% across both clinical sites (Mau et al., 2022).

#### 2.2.3 Maximal ATP production

^31^P MRS measures the rate of regeneration of phosphocreatine (PCr) after a short bout of exercise to quantify mitochondrial maximal ATP production (ATP_max_). This is a functional measure of in vivo ATP production via oxidative phosphorylation (Coen et al., 2013). In SOMMA, participants were instructed to lie in a supine position with their lower leg strapped and right knee joint in 20°–30° of flexion. ^31^P spectra were collected in a 3 Tesla MR scanner (Siemens Medical System—Prisma at Pittsburgh site or Skyra at Wake Forest site) using a 12″ dual‐tuned, surface RF coil (PulseTeq, Limited) placed over the right distal vastus lateralis. Participants did two bouts of isometric knee extension against the resistance of an ankle strap. The first bout was as hard and fast as they could for 30 s, and the second was adjusted for the length of time of muscle contraction (18–36 s) based on the first bout to ensure 30%–50% PCr depletion which was needed to obtain a high signal‐to‐noise defining full PCr recovery without acidosis (PH < 6.8; Jubrias et al., 2003). PCr recovery rate after exercise until PCr returned to baseline levels was fit, and the time constant of the mono‐exponential fit (tau) was used to calculate ATP_max_ (Amara et al., 2008; Blei et al., 1993). Our previous study illustrated good reproducibility of ATP_max_ evidenced by a high correlation (*r* = 0.92) between same‐day repeat scans (Santanasto et al., 2015). In SOMMA, the mean coefficient of variation for duplicates of ATP_max_ measurement was 9.9% across both clinic sites (Mau et al., 2022).

### 2.3 Pittsburgh Performance Fatigability Index

Participants wore an ActiGraph GT9X (Link) accelerometer (ActiGraph LLC) on their nondominant wrist during the usual‐paced 400‐m walk. Triaxial raw accelerometer data were collected at a sampling frequency of 80 Hz. During the usual‐paced 400‐m walk, participants were instructed to complete the distance at their usual pace without overexerting themselves (20 m each, a total of 10 laps in an unobstructed long corridor with traffic cones on both ends; Simonsick et al., 2008).

Raw accelerometer data collected during the 400‐m walk were processed in R (Version 4.0) to calculate PPFI, shown as a ratio of comparing the area under an individual's observed cadence‐versus‐time trajectory during the walking task to a hypothetical area that would be observed in the absence of fatigue (i.e., if the participant sustained maximal cadence throughout the entire 400‐m walk; Figure S1). Individual‐level smoothed cadence trajectories were fitted using penalized regression splines. Specific details about the derivation of PPFI have been published (Qiao et al., 2022). Participants who completed the usual‐paced 400‐m walk within 6 min exhibited no performance fatigability during the walking task (i.e., negligible decline in cadence), thus were classified as PPFI = 0%. Higher PPFI score (range 0%–100%) indicates more severe performance fatigability. Sex‐ and task‐specific PPFI cut‐points that optimally discriminate usual gait speed were also used to categorize participants into three severity strata: (1) no performance fatigability (PPFI = 0%); (2) mild performance fatigability (0% < PPFI < 3.5% for women; 0% < PPFI < 5.4% for men); and (3) moderate‐to‐severe performance fatigability (PPFI ≥ 3.5% for women; PPFI ≥ 5.4% for men; Qiao et al., 2023).

### 2.4 Covariates

Age, self‐reported sex, race (White, Black, Asian, Native American/Alaskan Native, Native Hawaiian/Pacific Islander, multiracial, or unknown), and smoking status (current/former/never) were obtained from each participant using self‐administered questionnaires. Race was further binarized for analyses as White and Black, Indigenous, and People of Color. Height (Harpenden stadiometers; Dyfed, UK) without shoes and weight (digital scales) with light clothing were assessed. The Short Physical Performance Battery (SPPB) consisted of three components: a balance battery with side‐by‐side, semi‐tandem, and tandem positions; a 4‐m usual pace walk; and five timed repeated chair stands. Each component was scored 0 (unable to complete) to 4 (best), and a summary score was calculated, ranging from 0 to 12, with higher scores indicating better physical function (Guralnik et al., 1994). Multimorbidity (yes/no) included self‐reported physician‐diagnosed hypertension, diabetes, heart diseases (including heart attack, coronary and myocardial infarction), stroke, lung diseases (including chronic obstructive lung disease, chronic bronchitis, asthma, emphysema, and chronic obstructive pulmonary disease), osteoporosis, and arthritis. Physical activity was measured by total activity count (Wolff‐Hughes et al., 2014) with the ActiGraph GT9X (Link) during a 7‐day free‐living period. We instructed participants to wear the device on their nondominant wrist at all times except underwater for more than 30 min. Valid days were defined as ≥17 h of wear time during a 24‐h period (00:00 to 23:59 for the same date) for ≥3 days (clinic visit day was excluded).

### 2.5 Statistical analyses

Among those enrolled in SOMMA (*N* = 879, 76.3 ± 5.0 years old, 59% women), 808 participants wore an accelerometer during the usual‐paced 400‐m walk, the source data used to derive PPFI, our key outcome of interest. Due to unidentifiable walking patterns based upon visual inspection of the accelerometer data, three participants were further excluded. Among participants with PPFI scores (*n* = 805), max OXPHOS data were available for 688 participants, max ETS data were available for 562 participants, and ATP_max_ data were available for 742 participants. The missingness for mitochondrial respiration was primarily due to assay data not meeting minimal quality control criteria or acidic conditions for ATP_max_. Thus, the final analytical sample with PPFI score, at least one skeletal muscle energetics measure, and no missing values on covariates was 795. All analyses were performed using R (R Foundation for Statistical Computing, version 4.0). Alpha was set to 0.05, and two‐sided *p* values smaller than 0.05 were considered significant.

Descriptive characteristics of participants were reported as median [25th percentile, 75th percentile], mean ± standard deviation (SD), or frequencies (percentages) in the overall sample and by PPFI severity strata. Trends across PPFI severity strata were examined using Kruskal–Wallis analysis of variance (ANOVA) for non‐normally distributed continuous variables, ANOVA for normally distributed continuous variables, and chi‐square tests for categorical variables. To understand the representativeness of our analytical sample, we compared demographic characteristics and physical function between participants with a biopsy and our final sample.

First, we examined Spearman's correlations (r_s_) and used scatterplots to illustrate bivariate correlations between muscle energetics and PPFI. Then, we generated regression models to examine the associations between muscle energetics and PPFI with progressive covariate adjustments to account for established and potential confounders based on prior literature (Mau et al., 2022; Simonsick et al., 2014). All muscle energetics variables were standardized before analyses. Model 1 adjusted for technician/site, age, sex, and race; Model 2 further adjusted for height and weight; and Model 3 additionally adjusted for total activity count. Given that PPFI scores were bounded between 0 and 100 and right‐skewed, tobit regressions were applied with continuous PPFI score. Whereas for categorical PPFI severity strata, we used ordinal logistic regressions and examined the proportional odds ratio assumption using the Brant–Wald test. Given that previous studies found a higher prevalence of performance fatigability in women (Simonsick et al., 2014) and differential associations between skeletal muscle energetics and physical function by physical performance (Santanasto et al., 2016), we further examined interactions with continuous PPFI score and sex or SPPB score.

## 3 RESULTS

### 3.1 Participant characteristics

Participants (*N* = 795) were 76.4 ± 5.0 years old (range: 70–94), 58% women, and 86% White. Median PPFI score was 1.4% [0%, 2.9%], range of 0%–21.7%, with 283 (35.6%) participants having no performance fatigability, 400 (50.3%) participants having mild performance fatigability, and 112 (14.1%) participants having moderate‐to‐severe performance fatigability (Table 1). From lowest to highest PPFI severity strata, median max OXPHOS, median max ETS, and median ATP_max_ values were lower, all *p* trend < 0.001 (Table 1). Across PPFI severity strata, participants had slower usual gait speed, worse SPPB score, and lower total active count, all *p* trend ≤ 0.02 (Table 1). No differences were found across PPFI severity strata in terms of health conditions, except for arthritis (Table 1). Lastly, no differences were observed among participants with different skeletal muscle energetic measures (Table S1).

**TABLE 1:** Baseline characteristics of the total sample (*N* = 795) and stratified by Pittsburgh Performance Fatigability Index (PPFI) severity strata in the Study of Muscle, Mobility and Aging (SOMMA).

Characteristics	Total (*N* = 795)	No performance fatigability^a^ (*n* = 283)	Mild performance fatigability^a^ (*n* = 400)	Moderate‐to‐severe performance fatigability^a^ (*n* = 112)	*p* Trend
PPFI, 0%–100%	1.39 [0, 2.90]	0 [0, 0]	2.00 [1.27, 2.82]	5.38 [4.21, 6.42]	
Max OXPHOS, pmol/(s × mg)	56.9 [46.2, 69.5]	62.4 [52.8, 77.8]	54.1 [43.4, 65.1]	53.7 [44.1, 60.5]	<0.001
Missing	107 (13.5)	30 (10.6)	53 (13.3)	24 (21.4)	
Max ETS, pmol/(s × mg)	77.0 [64.9, 92.2]	81.5 [70.7, 97.7]	74.1 [62.1, 90.0]	70.4 [62.1, 85.6]	<0.001
Missing	233 (29.3)	55 (19.4)	135 (33.8)	43 (38.4)	
ATP_max_, mM/s	0.51 [0.44, 0.61]	0.55 [0.47, 0.67]	0.50 [0.43, 0.60]	0.47 [0.42, 0.54]	<0.001
Missing	53 (6.7)	28 (9.9)	21 (5.3)	4 (3.6)	
Age, years	76.4 ± 5.0	75.0 ± 3.8	77.1 ± 5.4	77.4 ± 5.5	<0.001
Sex, women	462 (58.1)	134 (47.3)	240 (60.0)	88 (78.6)	<0.001
Race, white	684 (86.0)	255 (90.1)	341 (85.3)	88 (78.6)	0.01
Height, cm	166.0 ± 9.8	168.2 ± 9.4	165.2 ± 10.0	163.0 ± 9.1	<0.001
Weight, kg	76.3 ± 15.3	74.7 ± 15.1	77.5 ± 15.6	76.5 ± 14.6	0.14
Body mass index, kg/m^2^	27.6 ± 4.6	26.2 ± 4.1	28.3 ± 4.7	28.7 ± 4.8	<0.001
400‐m gait speed, m/s	1.05 ± 0.18	1.23 ± 0.10	0.96 ± 0.11	0.89 ± 0.13	<0.001
Short Physical Performance Battery, 0–12	10.2 ± 1.8	11.1 ± 1.1	9.7 ± 1.8	9.3 ± 2.0	<0.001
Hypertension^b^	411 (51.7)	148 (52.3)	200 (50.0)	63 (56.3)	0.70
Diabetes^c^	123 (15.5)	32 (11.3)	70 (17.5)	21 (18.8)	0.37
Heart diseases^c^	56 (7.0)	18 (6.4)	32 (8.0)	6 (5.4)	0.74
Stroke^c^	21 (2.6)	9 (3.2)	6 (1.5)	6 (5.4)	0.41
Lung disease^c^	105 (13.2)	34 (12.0)	51 (12.8)	20 (17.8)	0.80
Osteoporosis^c^	140 (17.6)	47 (16.6)	64 (16.0)	29 (25.9)	0.30
Arthritis^c^	443 (55.7)	127 (44.9)	245 (61.3)	71 (63.4)	<0.001
Fall history^d^	222 (27.9)	67 (23.7)	124 (31.0)	31 (27.7)	0.54
Total activity count, 10,000 counts/d	198 ± 58	213 ± 59	191 ± 57	187 ± 55	<0.001

*Note*: All reported in median [25th percentile, 75th percentile], mean ± SD or *n* (%).

Abbreviations: ATP, adenosine triphosphate; ETS, electronic transfer system; OXPHOS, oxidative phosphorylation; PPFI, Pittsburgh Performance Fatigability Index.

^a^ No performance fatigability: PPFI = 0; mild performance fatigability: 0 < PPFI < 3.5 for women, and 0 < PPFI < 5.4 for men; moderate‐to‐severe performance fatigability: PPFI ≥ 3.5 for women and PPFI ≥ 5.4 for men.

^b^ Hypertension was classified by systolic blood pressure ≥130 mmHg or diastolic blood pressure ≥80 mmHg.

^c^ Diabetes and all following health conditions were self‐reported physician diagnoses. Heart diseases included heart attack or myocardial infarction, heart failure, or atrial fibrillation. Lung diseases included chronic obstructive lung disease, chronic bronchitis, asthma, emphysema, and COPD.

^d^ Fall history was self‐reported and asked as “During the past 12 months, have you fallen and landed on the floor or ground, or fallen and hit an object like a table or chair?”

### 3.2 Associations between skeletal muscle energetics and continuous Pittsburgh Performance Fatigability Index scores

**FIGURE 1:**
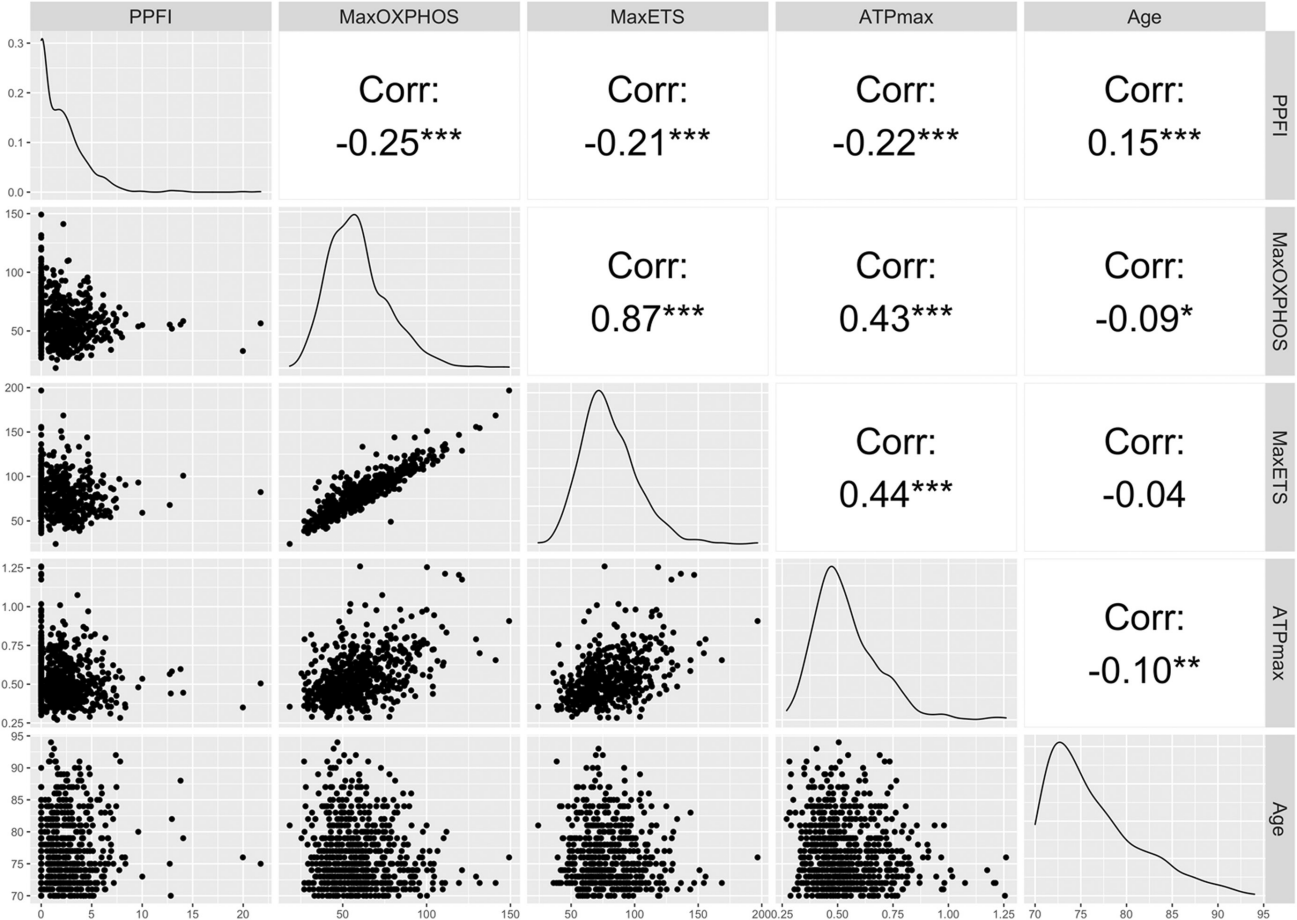
Spearman's correlations between skeletal muscle energetics, Pittsburgh Performance Fatigability Index (PPFI) score, and age in the Study of Muscle, Mobility and Aging (*N* = 795; there were *n* = 107 participants missing max OXPHOS, *n* = 233 missing max ETS, *n* = 53 missing ATP_max_).***p < 0.001, **p < 0.01, *p < 0.05. ATP, adenosine triphosphate; ETS, electronic transfer system; OXPHOS, oxidative phosphorylation; PPFI, Pittsburgh Performance Fatigability Index.

Among all, lower max OXPHOS (*r*_s_ = −0.25), max ETS (*r*_s_ = −0.21), and ATP_max_ (*r*_s_ = −0.22) were significantly correlated with higher PPFI score (Figure 1). In Tobit regression models, lower max OXPHOS, max ETS, and ATP_max_ were all significantly associated with higher PPFI score after adjustment for covariates (Table 2, Model 3). The partial *R* ^2^ for muscle energetics in Model 3 was the highest with max ETS (partial *R* ^2^ = 0.22), followed by ATP_max_ (partial *R* ^2^ = 0.06) and max OXPHOS (partial *R* ^2^ = 0.04; Table 2), indicating that max ETS explained considerably more variance in PPFI score than ATP_max_ and max OXPHOS. Note: Partial *R* ^2^ for max OXPHOS and ATP_max_ did not change even when the sample was restricted to only those with a max ETS measure (*n* = 562; data not shown).

**TABLE 2:** Associations of skeletal muscle mitochondrial energetics and Pittsburgh Performance Fatigability Index (PPFI) in older adults in the Study of Muscle, Mobility and Aging^a^.

	Model 1		Model 2		Model 3	
	*β* (95% CI)	Partial *R* ^2^	*β* (95% CI)	Partial *R* ^2^	*β* (95% CI)	Partial *R* ^2^
Max OXPHOS (1 SD = 18.4 pmol/(s × mg); *n* = 688)	0.65 (0.35, 0.94)	0.04	0.60 (0.30, 0.89)	0.04	0.55 (0.26, 0.84)	0.04
Max ETS (1 SD = 22.1 pmol/(s × mg); *n* = 562)	0.48 (0.17, 0.79)	0.23	0.43 (0.12, 0.74)	0.22	0.39 (0.09, 0.70)	0.22
ATP_max_ (1 SD = 0.15 mM/s; *n* = 742)	0.66 (0.39, 0.93)	0.06	0.65 (0.38, 0.92)	0.06	0.54 (0.27, 0.81)	0.06

Abbreviations: ATP, adenosine triphosphate; ETS, electronic transfer system; OXPHOS, oxidative phosphorylation; PPFI, Pittsburgh Performance Fatigability Index.

^a^ Model 1 adjusted for technician/site, age, sex, race; Model 2 further adjusted for height and weight on top of Model 1; Model 3 additionally adjusted for total activity count from wrist‐worn ActiGraph GT9X.

After full adjustment, each 1 SD lower max OXPHOS (18.4 pmol/(s × mg)), max ETS (22.1 pmol/(s × mg)), and ATP_max_ (0.15 mM/s) were associated with 0.55 (95% CI: 0.26, 0.84), 0.39 (95% CI: 0.09, 0.70), and 0.54 (95% CI: 0.27, 0.81) higher PPFI score, respectively (Table 2). No sex interactions were found (all *p* interaction > 0.05). The association for max OXPHOS with PPFI significantly varied by physical function (*p* interaction = 0.046); interactions were not significant for max ETS (*p* interaction = 0.34) and ATP_max_ (*p* interaction = 0.10). Thus, when stratified by physical function (better: SPPB score ≥10 vs. worse: SPPB < 10), lower max OXPHOS was only associated with higher PPFI score for participants with better physical function (*β* = 0.52 [95% CI: 0.20, 0.85] for SPPB ≥ 10 and *β* = 1.56 [95% CI: −0.44, 0.76] for SPPB < 10).

### 3.3 Associations between skeletal muscle energetics and Pittsburgh Performance Fatigability Index severity strata

**FIGURE 2:**
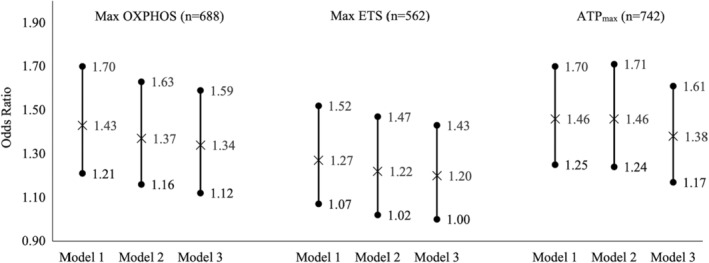
Associations of skeletal muscle energetics and Pittsburgh Performance Fatigability Index (PPFI) severity strata in older adults in the Study of Muscle, Mobility and Aging (SOMMA). ATP, adenosine triphosphate; ETS, electronic transfer system; OXPHOS, oxidative phosphorylation; PPFI, Pittsburgh Performance Fatigability Index. No performance fatigability: PPFI = 0; mild performance fatigability: 0 < PPFI < 3.5 for women, and 0 < PPFI < 5.4 for men; moderate‐to‐severe performance fatigability: PPFI ≥ 3.5 for women and PPFI ≥ 5.4 for men. Model 1 adjusted for technician/site, age, sex, race. Model 2 further adjusted for height and weight on top of Model 1. Model 3 additionally adjusted for total activity count from wrist‐worn ActiGraph GT9X.

In ordinal logistic regression models, lower max OXPHOS, max ETS, and ATP_max_ yielded greater odds of being in a higher PPFI severity stratum before adjustment for total activity count (Figure 2, Model 2). However, max ETS was no longer associated with PPFI severity strata after adjusting for total activity count (odds ratio (OR) = 1.20 [95% CI: 1.00, 1.43]) (Figure 2, Model 3). Each 1 SD lower max OXPHOS and lower ATP_max_ were associated with 34% (95% CI: 1.12, 1.59) and 38% (95% CI: 1.17, 1.61) greater odds of being in a higher PPFI severity stratum, respectively (Figure 2, Model 3). Similar to the analyses with continuous PPFI score, only max OXPHOS showed differential associations with PPFI severity strata when stratified with physical function. Specifically, each 1 SD lower max OXPHOS was associated with 32% greater odds of being in a higher PPFI severity stratum among participants with better physical function (OR = 1.32 [95% CI: 1.07, 1.60] for SPPB ≥ 10 and OR = 1.22 [95% CI: 0.68, 1.67] for SPPB < 10).

## 4 DISCUSSION

Lower skeletal muscle energetics, particularly max OXPHOS, and ATP_max_ were significantly associated with higher PPFI score and greater odds of being in a higher PPFI severity stratum, whereas lower max ETS was only significantly associated with higher PPFI score, but not PPFI severity after adjustment for total activity count. To our knowledge, this is the first study to evaluate the associations between muscle energetics both in vivo and ex vivo with performance fatigability in a large cohort of older adults. In addition, the inverse associations between ATP_max_ with performance fatigability align with previous studies examining ATP_max_ and perceived fatigability (Liu et al., 2021; Santanasto et al., 2015). Furthermore, given that max ETS (22%) explained more of the variance in performance fatigability than max OXPHOS (4%) and ATP_max_ (6%), it implies that there might be a differential impact on performance fatigability across various aspects of mitochondrial function. Although developing lifestyle or pharmaceutical interventions that can differentially target specific muscle energetics may be challenging, our findings suggest that improving mitochondrial function or content in general would be beneficial to reduce performance fatigability.

Max ETS is an assessment of the maximal capacity to transport electrons when membrane potential has collapsed due to uncoupling and is not limited by the capacity of the phosphorylation system. It is important to note that max ETS measured ex vivo is not physiologically evident in human tissue per se because completely uncoupled respiration does not occur naturally in skeletal muscle (Gnaiger et al., 2019). However, max ETS is a valuable, focused assessment of electron transport system function, a fundamental constituent of mitochondrial energetics, under standardized experimental conditions. Studies have indicated that ETS dysfunction was associated with lower mitochondrial energy production and higher reactive oxygen species (ROS). It also corresponds to onset and development of obesity, aging, and pathology in many organ systems including cardiovascular diseases (Nolfi‐Donegan et al., 2020). Thus, lower max ETS not only indicates less oxygen flux to support membrane potential and OXPHOS, but also suggests potential decrements due to aging or chronic health conditions. Given that performance fatigability has been proposed as a manifestation of diminishing reserve between daily energy utilization and one's energy capacity (Alexander et al., 2010), it was expected that lower max ETS was associated with higher PPFI score. A caveat is that max ETS capacity is also considered a proxy measure for mitochondrial content (e.g., mtDNA count). Future studies are needed to examine markers of mitochondrial content to confirm the physiological effect of ETS capacity per se on performance fatigability, rather than the effect reflecting differences in mitochondrial content. An ancillary study in SOMMA is currently analyzing mtDNA copy number (a marker of mitochondrial content) from collected biopsies.

Max OXPHOS and ATP_max_ showed similar strong associations with continuous PPFI score and PPFI severity strata. There are a few potential explanations for their similar associations with performance fatigability. First, both max OXPHOS and ATP_max_ reflect ATP production via oxidative phosphorylation, and they were moderately correlated with each other in our study (*r*_s_ = 0.43). Second, as mutations in mtDNA accumulate with aging and diseases, function and structure of OXPHOS complexes can be altered and lead to skeletal muscle energetics dysfunction which accelerates ROS generation (Bua et al., 2006; Wallace, 2010). Thus, lower max OXPHOS as measured ex vivo and lower ATP_max_ as measured in vivo may lead to a lower rate of ADP phosphorylation and increased ROS accumulation, which in turn limits skeletal muscle energy to maintain a faster walking speed potentially resulting in higher performance fatigability. Third, the in vivo ATP_max_ measure is dependent on more physiological factors beyond OXPHOS, such as oxygen delivery, muscle perfusion, and neuromuscular activation (Casas et al., 2014; Hochachka & McClelland, 1997). Also, our results concur with previous findings in terms of associations between ATP_max_ and perceived fatigability (Liu et al., 2021; Santanasto et al., 2015). Collectively, our results imply that therapeutics aimed at directly improving ATP production at the mitochondria level or peripheral physiological factors that influence oxygen delivery could both potentially mitigate fatigability and lessen susceptibility to disability among older adults.

In the current study, skeletal muscle energetics were standardized when modeling. To facilitate a better understanding of the magnitude of the associations, we provide context as to what 1 SD of muscle energetics means in regard to age and cardiorespiratory fitness. In general, comparing adults in their 30s to adults in their 70s, OXPHOS and ATP production were lower by 30%–50% (Boffoli et al., 1994; Cooper et al., 1992; Trounce et al., 1989). For example, younger adults (*n* = 9, aged 25–48 years) had ATP_max_ of 6.8 ± 0.6 mM, while older adults (*n* = 40, aged 65–80 years) had ATP_max_ of 5.9 ± 0.2 mM (Conley et al., 2000), similar to our data in SOMMA. Thus, 1 SD of ATP_max_ in SOMMA approximated the difference observed with two‐decade difference in age. While this may seem like a substantial change, a study of 24 weeks of either endurance training or resistance training among 40 older adults (mean age = 69.2 ± 0.6 years old) both showed improvement in ATP_max_ from 0.54 to 0.70 mM/s and 0.63 to 0.99 mM/s, respectively (Jubrias et al., 2001), which approximately equaled to 1 SD ATP_max_ in SOMMA. Prospective longitudinal data are needed to estimate annual changes in muscle energetics to better contextualize meaningful differences in skeletal muscle energetics.

Additionally, we found that only max OXPHOS was associated with performance fatigability among participants with better physical function but not among participants with worse physical function. Yet, no differential associations were observed for max ETS nor ATP_max_. Although we need more studies to confirm the effect modification by physical function in relation to muscle energetics and performance fatigability, our findings could be better understood by a previous study that found ATP_max_ was only associated with time to walk 400 m among those with better physical function (Santanasto et al., 2016). Santanasto et al. (2016) postulated that those with higher levels of ATP_max_ and longer 400‐m walk completion times might have biomechanical inefficiency manifested as a lower mitochondrial efficiency (P/O) and higher energetic cost of walking. We will further investigate the plausibility of these postulations in SOMMA in future work, in order to understand the interrelationships between skeletal muscle energetics, energetic cost of walking, and performance fatigability among lower‐functioning older adults.

There are several strengths and some limitations of our study. First, SOMMA had both ex vivo assessment of skeletal muscle mitochondrial respiration (including max OXPHOS and max ETS) and in vivo assessment of ATP_max_, in a large sample of older women and men ranging from 70 to 94 years old, providing comprehensive skeletal muscle energetics. Given our observed differences in PPFI score and muscle energetics values between white versus non‐white subgroups in SOMMA, the generalizability of our results might be limited for more diverse race/ethnicity populations, due to the largely white sample. Yet, our study participants had comparable muscle energetics values with the same age group demonstrated in other studies (Berg et al., 2020; Coen et al., 2013). Third, we excluded participants with missingness for certain muscle energetic measures in their relevant analyses. However, we found no significant differences in covariates among participants with different muscle energetic measures and no significant differences among our final analytical sample versus excluded participants. Lastly, given that a usual‐paced 400‐m walk is a less strenuous task, we may have observed lower PPFI scores and found more conservative associations between muscle energetics and fatigability. Nonetheless, PPFI is validated for use in conjunction with a usual‐paced 400‐m walk with good validity (Qiao et al., 2023).

In conclusion, we found that lower skeletal muscle energetics were associated with more severe performance fatigability. Our understanding of skeletal muscle energetics and performance fatigability may inform new directions for lifestyle interventions and potential mitochondrial medicine aimed at reducing fatigability, improving functional performance, and preventing mobility disability.

## AUTHOR CONTRIBUTIONS

Drs. Qiao and Glynn had full access to all of the data for the study and take responsibility for the integrity of the data and accuracy of the data analyses. All authors: interpretation of data, critical revision of manuscript for important intellectual content. All authors read and approved the submitted manuscript.

## ACKOWLEDGEMENTS

We acknowledge all the staff and investigators, and we appreciate all the SOMMA participants.

## FUNDING INFORMATION

The Study of Muscle, Mobility and Aging is supported by funding from the National Institute on Aging (AG 059416). Study infrastructure support was funded in part by NIA Claude D. Pepper Older American Independence Centers at University of Pittsburgh (P30 AG024827) and Wake Forest University (P30 AG021332) and the Clinical and Translational Science Institutes, funded by the National Center for Advancing Translational Science at Wake Forest University (UL1 0TR001420). K.D.M. was supported by the Pittsburgh Epidemiology of Aging Training Program (NIA T32 AG000181).

## CONFLICT OF INTEREST STATEMENT

None declared.

## Supporting information


Appendix S1.

## Data Availability

The data that support the findings of this study are available at http://sommaonline.ucsf.edu.
