# Supplementary material for: Associations between skeletal muscle energetics and accelerometry‐based performance fatigability: Study of Muscle, Mobility and Aging
Source: Aging Cell. 2023 Oct 16;23(6):e14015. doi: 10.1111/acel.14015 (PMC11166367; doi:10.1111/acel.14015)
Supplement: Supplementary file 1 — Appendix S1. [file ACEL-23-e14015-s001.docx]

**Associations between Skeletal Muscle Energetics and Accelerometry-based Performance Fatigability: Study of Muscle, Mobility and Aging (SOMMA)**

**AUTHORS:** Yujia (Susanna) Qiao^1^, PhD, ScM; Adam J. Santanasto^1^, PhD, MPH; Paul M. Coen^2^, PhD; Peggy M. Cawthon^3,4^, PhD; Steve R. Cummings^3^, PhD; Daniel E. Forman^5^, MD; Bret H. Goodpaster^2^, PhD; Jaroslaw Harezlak^6^, PhD; Marquis Hawkins^1^, PhD; Stephen B. Kritchevsky^7^, PhD; Barbara J. Nicklas^7^, PhD; Frederico G.S. Toledo^8^, DM; Pamela E. Toto^9^, PhD; Anne B. Newman^1^, MD, MPH; and Nancy W. Glynn^1^, PhD

**Supplemental Table 1.** Baseline characteristics stratified by skeletal muscle energetics measures in the Study of Muscle, Mobility and Aging (SOMMA)

| Characteristics | Total  (N=795) | Had max OXPHOS (n=688) | Had max ETS (n=562) | Had ATP_max_  (n=742) | p-value |
| --- | --- | --- | --- | --- | --- |
| PPFI, 0-100% | 1.39 [0, 2.90] | 1.31 [0, 2.84] | 1.12 [0, 2.83] | 1.46 [0, 2.91] | 0.50 |
| PPFI severity strata^1^ |  |  |  |  | 0.44 |
| No performance fatigability | 283 (35.6) | 253 (36.8) | 228 (40.6) | 255 (34.4) |  |
| Mild performance fatigability | 400 (50.3) | 347 (50.4) | 265 (47.2) | 379 (51.1) |  |
| Moderate-to-severe performance  fatigability | 112 (14.1) | 88 (12.8) | 69 (12.3) | 107 (14.6) |  |
| Max OXPHOS, pmol/(s*mg) | 56.9 [46.2, 69.5] | 56.9 [46.2, 69.5] | 58.6 [48.2, 72.2] | 56.6 [46.0, 69.6] | 0.28 |
| Missing | 107 (13.5) | 0 | 0 | 107 (5.4) |  |
| Max ETS, pmol/(s*mg) | 77.0 [64.9, 92.2] | 77.0 [64.9, 92.2] | 77.0 [64.9, 92.2] | 77.2 [64.8, 92.7] | 1.00 |
| Missing | 233 (29.3) | 126 (18.3) | 0 | 232 (31.3) |  |
| ATP_max_, mM/sec | 0.51 [0.44, 0.61] | 0.52 [0.44, 0.62] | 0.51 [0.44, 0.62] | 0.51 [0.44, 0.62] | 0.90 |
| Missing | 53 (6.7) | 53 (7.7) | 52 (9.3) | 0 |  |
| Age, years | 76.4 ± 5.0 | 76.4 ± 5.0 | 76.1 ± 4.7 | 76.4 ± 5.1 | 0.68 |
| Sex, women | 462 (58.1) | 377 (54.8) | 305 (54.3) | 437 (58.9) | 0.31 |
| Race, white | 684 (86.0) | 596 (86.6) | 488 (86.8) | 638 (86.0) | 0.97 |
| Height, cm | 166.0 ± 9.8 | 166.3 ± 9.9 | 166.4 ± 9.9 | 166.0 ± 981 | 0.87 |
| Weight, kg | 76.3 ± 15.3 | 76.4 ± 15.6 | 76.6 ± 15.4 | 76.6 ± 15.5 | 0.99 |
| Body mass index, kg/m^2^ | 27.6 ± 4.6 | 27.5 ± 4.6 | 27.6 ± 4.6 | 27.7 ± 4.7 | 0.87 |
| 400m gait speed, m/s | 1.05 ± 0.18 | 1.23 ± 0.10 | 0.96 ± 0.11 | 0.89 ± 0.13 | <.001 |
| Short Physical Performance Battery, 0-12 | 10.2 ± 1.8 | 10.2 ± 1.8 | 10.3 ± 1.7 | 10.1 ± 1.8 | 0.10 |
| Hypertension^2^ | 411 (51.7) | 362 (52.6) | 293 (52.1) | 391 (52.7) | 1.00 |
| Diabetes^3^ | 123 (15.5) | 102 (14.8) | 81 (14.4) | 114 (15.4) | 0.99 |
| Heart diseases^3^ | 56 (7.0) | 52 (7.6) | 36 (6.4) | 54 (7.3) | 0.89 |
| Stroke^3^ | 21 (2.6) | 17 (2.5) | 12 (2.1) | 21 (2.8) | 0.98 |
| Lung disease^3^ | 105 (13.2) | 84 (12.2) | 72 (12.8) | 101 (13.6) | 0.98 |
| Osteoporosis^3^ | 140 (17.6) | 117 (17.0) | 93 (16.5) | 134 (18.1) | 0.99 |
| Arthritis^3^ | 443 (55.7) | 378 (54.9) | 305 (54.3) | 414 (55.8) | 0.99 |
| Fall history^4^ | 222 (27.9) | 188 (27.3) | 148 (26.3) | 207 (27.9) | 0.99 |
| Total activity count, counts/d | 1984111 ± 583442 | 1973814 ± 580256 | 1977269 ± 581873 | 1988422 ± 579889 | 0.97 |

All reported in median [25^th^ percentile, 75^th^ percentile], mean ± SD or n (%). Abbreviations: PPFI = Pittsburgh Performance Fatigability Index; OXPHOS = oxidative phosphorylation; ETS = electronic transfer system; ATP = adenosine triphosphate

^1^ No performance fatigability: PPFI=0; mild performance fatigability: 0<PPFI<3.5 for women, and 0<PPFI<5.4 for men; moderate-to-severe performance fatigability: PPFI ≥3.5 for women and PPFI ≥5.4 for men

^2^ Hypertension was classified by systolic blood pressure ≥130 mmHg or diastolic blood pressure ≥80 mmHg

^3^ Diabetes and all following health conditions were self-reported physician diagnoses. Heart diseases included heart attack or myocardial infarction, heart failure, or atrial fibrillation. Lung diseases included chronic obstructive lung disease, chronic bronchitis, asthma, emphysema, and COPD

^4^ Fall history was self-reported and asked as “During the past 12 months, have you fallen and landed on the floor or ground, or fallen and hit an object like a table or chair?”


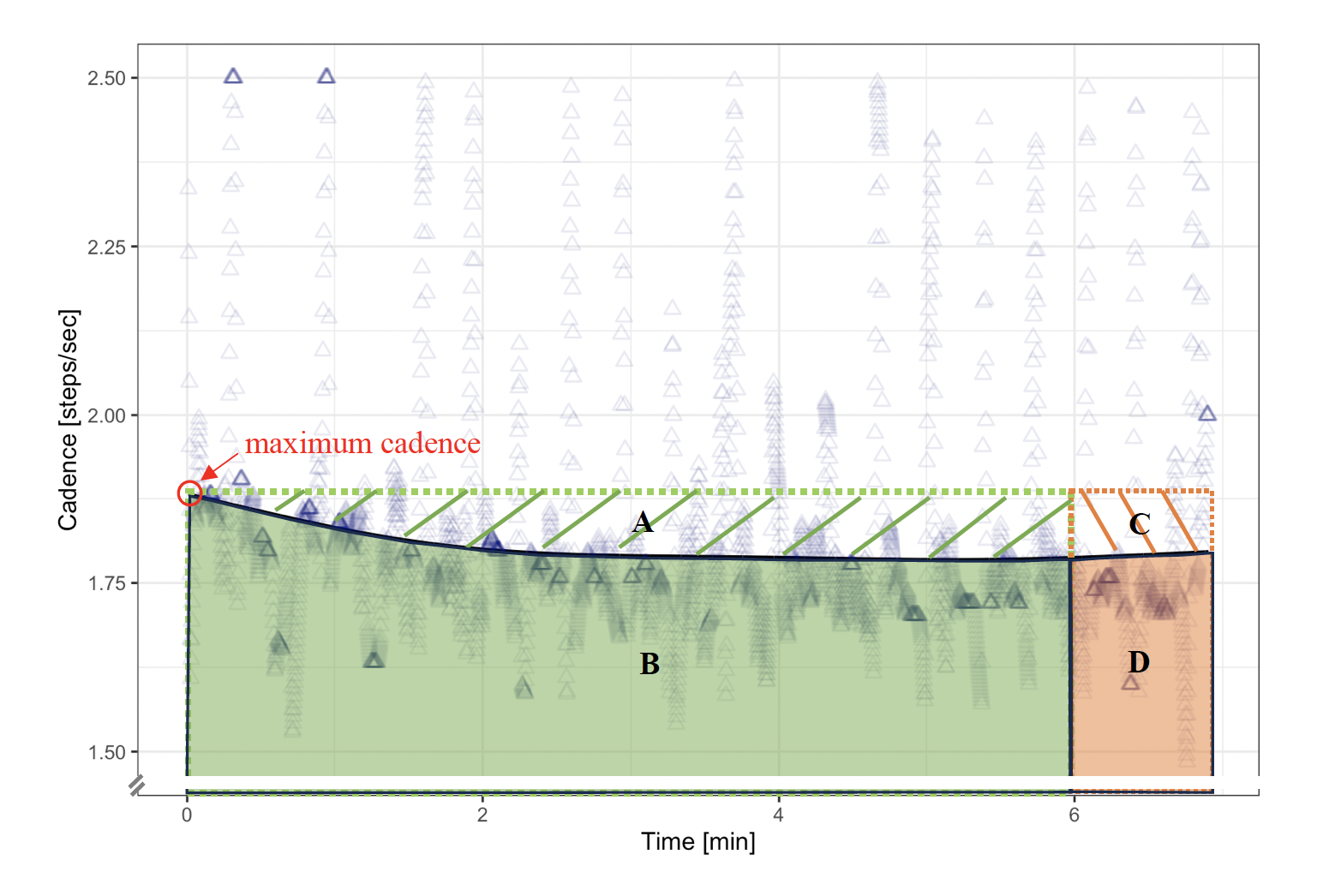


**Supplemental Figure 1.** Illustrative examples of an individual’s PPFI score from a usual-paced 400m walk. The blue triangles represent raw cadence estimates; the black line represents the individual-smoothed cadence trajectory. A = dashed green area, B = shaded green area, C = dashed orange area, D = shaded orange area. The PPFI equation is: [(A / A+B) * (B / B+D) + (C / C+D) * (D / B+D)] * 100%.
